# Complete Left-Atrial Lesion Set versus Pulmonary Vein Isolation Only in Patients with Paroxysmal AF Undergoing CABG or AVR

**DOI:** 10.3390/medicina58111607

**Published:** 2022-11-07

**Authors:** Yalin Yildirim, Johannes Petersen, Ali Aydin, Yousuf Alassar, Hermann Reichenspurner, Simon Pecha

**Affiliations:** 1 Department of Cardiovascular Surgery, University Heart Center Hamburg, 20246 Hamburg, Germany; 2 DZHK (German Centre for Cardiovascular Research), Partner Site Hamburg/Kiel/Lübeck, 20246 Hamburg, Germany; 3 Heart Center Bremen-Kardiologic-Angiologic Practice (KAP) Bremen, 28277 Bremen, Germany

**Keywords:** atrial fibrillation, surgical ablation, arrhythmia surgery, pulmonary vein isolation, left-atrial ablation

## Abstract

*Background and Objectives*: In patients with paroxysmal atrial fibrillation (AF) undergoing CABG or aortic valve surgery, many surgeons are not willing to open the left atrium to perform a complete left-sided Cox-Maze lesion set. Pulmonary vein isolation (PVI) is often favored in those patients. We investigated the outcome of patients with isolated pulmonary vein isolation compared to those receiving an extended left atrial (LA) lesion set. *Materials and Methods*: Between 2003 and 2016, 817 patients received concomitant surgical AF ablation in our institution. A total of 268 patients with paroxysmal AF were treated by surgical ablation concomitant to AVR or CABG. Of those, 86 patients underwent a complete left-sided lesion set, while 182 patients were treated with an isolated pulmonary vein isolation. The primary endpoint was freedom from atrial fibrillation at 12 months’ follow-up. *Results:* There were no statistically significant differences regarding baseline characteristics. No major ablation-related complications were observed in any of the groups. In the PVI group, three patients (1.6%) had an intraoperative stroke, while two (2.3%) patients experienced a stroke in the LA ablation group (*p* = 0.98). In-hospital mortality was 3.4% in the PVI group, and 2.8% in the extended LA group (*p* = 0.33). Freedom from AF at 12 months’ follow-up was 76% in the extended LA ablation group and 70% in the PVI group, showing no statistically significant difference (*p* = 0.32). *Conclusion:* Surgical AF ablation concomitant to CABG or AVR in patients with paroxysmal AF is safe and effective. There was no statistically significant difference between the compared lesion sets in terms of freedom from AF, survival or stroke rate after 12 months.

## 1. Introduction

Atrial fibrillation (AF) is the most common arrhythmia and is associated with thromboembolic events, including stroke, and is even associated with an increased mortality [1,2]. Furthermore, it leads to heart failure and contributes to an increased number of hospitalizations [1,2]. Concomitant surgical AF ablation has been shown to reduce AF in retrospective as well as in prospective randomized trials [3,4,5,6,7]. In retrospective studies and prospective registries a survival benefit has also been shown for patients with AF undergoing concomitant surgical ablation [5,8,9]. Therefore, concomitant AF ablation is recommended in guidelines and consensus statements [10,11].

The initial Cox Maze procedure, which included the cut-and-sew technique, has been modified to the Cox Maze III procedure, which has been the gold standard for surgical ablation for many years [12]. Nevertheless, due to the complexity of this procedure, it has only been used by a few surgeons. When the cut- and sew technique was replaced by the creation of thermal lesions, that simplified the procedure and resulted in a wide-spread application as a Cox MAZE IV procedure. Over the years, many modifications of the initial Cox Maze IV lesion set have been used with varying success rates. In patients with paroxysmal AF, the pulmonary veins are the most common trigger of arrhythmias. It has been shown by Haissaguerre that in patients with paroxysmal AF without structural heart disease, up to 94% of the AF triggers originate from the pulmonary veins [13]. Although the concomitant surgical AF ablation population is differing from the collective described by Haissaguerre, in those with paroxysmal AF, the pulmonary veins are very likely to be the origin of triggers for AF in most of the cases. In procedures where the atria are not routinely opened for the surgical procedure, such as in CABG or AVR cases, many surgeons are not willing to conduct an atriotomy to perform a complete left-atrial- or even a biatrial ablation. For this patient collective with paroxysmal AF, there is no clear evidence if there is an advantage of a complete left- atrial lesion set over a pulmonary vein isolation only. We therefore investigated whether there is a difference in ablation success and procedural outcome for patients receiving a complete left-atrial lesion set or an isolated PVI in patients undergoing CABG or AVR.

## 2. Materials and Methods

Between January 2003 and 2016, 817 patients were treated with concomitant surgical ablation in our institution. A total of 268 patients with paroxysmal AF were treated concomitant to aortic valve surgery and/or coronary artery bypass grafting and were included in this retrospective data analysis. Patients treated with unipolar radiofrequency (*n* = 26) were excluded from this analysis due to the inferiority of unipolar radiofrequency as an energy source. IRB approval was obtained (Ethikkommission der Ärztekammer Hamburg 2020-10183). An isolated pulmonary vein isolation (PVI) has been used in 182 patients, while a complete left-atrial lesion set was used in 86 patients. The choice of the lesion set was left to the discretion of the operator. The complete left-atrial lesion set included a box lesion as well as left atrial appendage- and isthmus isolation. A retrospective single center data analysis was performed. The results and outcomes of patients with PVI was compared to patients receiving a complete left atrial lesion set. Applied energy sources included argon-based cryoablatation (CryoICE Cryo-ablation probe, Atricure Inc. West Chester, OH, USA; Cardioblate CryoFlex Surgical Ablation Probe, Medtronic Inc., Minneapolis, MN, USA) in 79 patients and bipolar ablation (Cardioblate BP2 device and Cardioblate Surgical Ablation System Generator, Medtronic Inc., Minneapolis, MN, USA or Atricure Isolator Synergy Access Clamp EMT1, Atricure Inc. West Chester, OH, USA) in 189 patients.

### 2.1. Follow-Up

Follow-up was conducted by either implantable loop recorder (ILR) interrogation (*n* = 67) or 24-h Holter ECG (*n* = 227) in our clinic 3 and 12 months post-surgery. Follow-up methods were equally distributed between groups (PVI group: ILR *n* = 48 (26.4%); extended LA group: ILR *n* = 19 (22.1%), *p* = 0.42. AF recurrence was defined by AF Burden >0.5% in ILR interrogation or duration of AF episode >30 s in 24 h- Holter- ECG. The discharge rhythm results were obtained by 12 lead ECG. The anticoagulation regimen was maintained for three months postoperative in all patients and then adapted according to CHA_2_DS_2_-VASc score. In patients without contraindications, amiodarone was used as an antiarrhythmic drug therapy, otherwise other class I or III antiarrhythmic drugs were used for at least three months postoperative and then adapted according to either ILR- or 24 h ECG rhythm results.

### 2.2. Statistical Analysis

All statistical analyses were done with SPSS statistical software version 28.0 (SPSS Inc., Chicago, IL, USA) Continuous values are displayed as mean ± standard deviation and were compared with a Students *t*-test if appropriate; otherwise a Mann Whitney test was used. Categorical variables are shown as frequency and percentages and were compared using the chi square test or Fishers exact test (<5 counts per cell) as appropriate. A *p*-value of less than 0.05 was considered statistically significant.

## 3. Results

### 3.1. Patient Characteristics

Baseline patient characteristics are shown in Table 1. There were no statistically significant differences between the groups. Mean patient age was 66.0 ± 8.6 years in the left atrial group and 67.4 ± 6.5 in the PVI group. Mean LA diameter was 53.2 ± 6.7% in patients with left-atrial lesion set and 52.5 ± 6.1% in PVI group. Mean left ventricular ejection fraction (LVEF) was 54.3 ± 6.5% for patients with a left-atrial lesion set and 54.8 ± 7.9 mm in persons with PVI only. Mean duration of AF was 3.0 ± 2.3 and 2.7 ± 3.5 years for patients with left-atrial lesion set and PVI only, respectively.

### 3.2. Procedural Data

Performed surgical procedures included isolated coronary artery bypass grafting (CABG) in 101 patients in the PVI group and 44 patients in the extended LA group. Aortic valve replacement was performed in 48 patients in the PVI group and 24 patients in the LA group, respectively. Combined CABG and AVR surgery was conducted in 33 patients of the PVI group and 18 patients with an extended LA ablation.

Mean cross-clamp time was 71 ± 8 min in the PVI group and 89 ± 9 min in the extended LA group, corresponding to a significantly longer cross-clamp time in patients receiving a complete LA lesion set (*p* < 0.001).

There were no major ablation-related complications in any of the patients. No intraoperative death was observed. In-hospital mortality was 3.4% in the PVI group and 2.8% in the extended LA ablation group (*p* = 0.33). The one-year survival rate was 94.7% in the PVI group and 93.5% in the extended LA group, without significant differences between groups. In three (1.6%) patients in the PVI group, a perioperative stroke occurred, while two (2.3%) patients in the extended LA group experienced a stroke (*p* = 0.96).

Permanent Pacemaker implantation rates were 5.0% and 6.4% in PVI- and the extended LA group, without statistically significant differences (*p* = 0.67). During follow-up, two patients in the PVI only group experienced left-atrial flutter, while none of the patients receiving an extended left atrial lesion set had LA flutter episodes. All patients with atrial flutter were successfully treated by catheter ablation.

### 3.3. Rhythm Results

**Figure 1:**
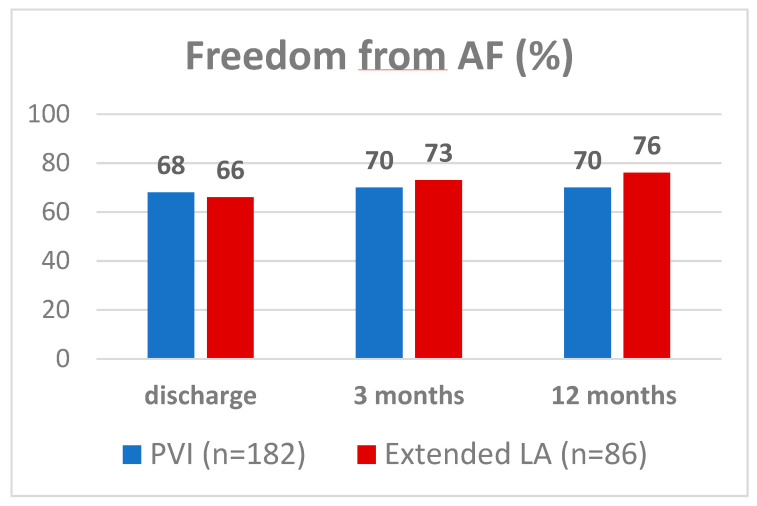
Rate of freedom from AF during follow up.

Patients received 24 h Holter-ECG or ER interrogation at 12 months’ follow-up. There were no statistically significant differences between groups regarding freedom from AF rate at discharge (PVI 68% vs. LA: 66% *p* = 0.22). Furthermore, at the three months’ follow-up, there was no significant difference regarding freedom from AF rate between the PVI and the left-atrial group (PVI: 70% vs. LA: 73% *p* = 0.54). One-year follow-up showed freedom from AF rate of 76% in patients with left-atrial ablation compared to 70% in the group of patients with PVI only, showing no statistically significant difference (*p* = 0.32) Figure 1. The rate of freedom from AF after one year off antiarrhythmic drugs was also comparable between groups (LA: 70% vs. PVI: 64% *p* = 0.49).

## 4. Discussion

In this study, comparing a complete left atrial lesion set vs. PVI only in patients with paroxysmal AF undergoing CABG or AVR, we have shown that there is no statistically significant difference in rhythm outcome at one-year follow-up. Furthermore, there were no differences regarding complication- and survival rates between groups.

In an pioneering electrophysiological study by Haissaguerre et al. in 1998, it has been shown that in stand-alone paroxysmal AF, the triggers initiating AF are mostly originating from the pulmonary veins [13]. Since then, in catheter-based interventional ablation, the pulmonary veins are the main target for AF ablation [14,15]. In surgical AF ablation, the Cox-Maze procedure, first performed by James Cox in 1987, has been the method of choice for several years, with excellent results. The Cox Maze IV ablation has been shown to be a safe and effective treatment method in prior publications [15]. However, the Cox Maze procedure necessitates an opening of both atria for ablation [12]. In patients without opening of the atria, in clinical reality, many surgeons are reluctant to open the atria for a complete biatrial lesion set. Several modifications of the biatrial lesion set have been published throughout the years [16,17]. Especially in patients with paroxysmal AF, a left-atrial lesion set- or an isolated PVI are alternatives, which are frequently used [4,18,19,20]. Our rhythm results for the PVI as well as the extended LA group are in line with previous published studies. Results for freedom from AF off antiarrhythmic drugs in patients with PVI only range between 51% and 75%, and in patients with the extended LA Lesion set the reported results are between 62% and 76%. In CABG or AVR cases, many surgeons are reluctant to open the left atrium for surgical ablation, as it adds cross-clamp- and bypass time to the procedure. The remaining question is whether this addition of a complete left-atrial lesion set results in an improved rhythm outcome. In recent years, only a few studies comparing the left-atrial lesion set vs. PVI only have been published, with conflicting results. In a recently published randomized controlled trial of surgical AF ablation concomitant to mitral valve surgery, an isolated PVI achieved similar results as a biatrial lesion set [7]. In contrast to these results, Soni et al. [21] have shown that a complete left-atrial lesion set leads to significantly higher rates of freedom from AF in comparison to PVI only. They found freedom from AF rates of 57% in the PVI group and 76% in the extended LA group (*p* < 0.001). However, in this study, patients with persistent- or longstanding persistent AF were also included. In those patients, with non-paroxysmal AF, atrial fibrillation might be more often initiated from triggers outside of the pulmonary veins. Here, macro-reentrant circuits from all over the atria might contribute to initiation and the perpetuation of atrial fibrillation.

Therefore, a more extensive ablation with a complete left-atrial lesion set or even a biatrial ablation might be necessary to achieve satisfactory results and to eliminate the non-pulmonary vein triggers and rotors. Furthermore, in patients with structural mitral valve disease, atrial fibrillation might originate from the left atrial wall itself. This might be related to the increased left atrial volume and pressure caused by the mitral regurgitation.

The type of atrial fibrillation has an effect on the outcome of patients. In a recent publication by Nuzzi and colleagues in a large cohort of patients with DCM, it was shown that the type of AF influences the outcome. Patients with permanent AF had a significantly worse outcome compared to patients with paroxysmal or persistent AF [22].

In our investigation, which was limited to patients with paroxysmal AF and without mitral valve disease, an isolated PVI achieved comparable rhythm results in the time frame of 12 months. In future, a longer follow-up might be interesting to compare the results in the long-term run and to draw definite conclusions concerning rhythm results over time. In our study, the use of ablation energies was not equally distributed between groups. In a group of patients receiving PVI, most of them were treated with bipolar radiofrequency while the majority of patients receiving the extended left-atrial lesion set were treated with cryoablation. This might have influenced the rhythm results in our study. However, in our own experience, as well as in other previously published papers, no differences in efficacy between cryoablation and bipolar radiofrequency ablation were observed.

The statistically significant longer cross-clamp time, which was observed in the extended LA group, had no influence on postoperative outcome. Both lesion sets were safe and feasible, with a low number of peri-procedural complications and similar survival-rates between groups during one year follow-up. Furthermore, the rate of permanent pacemaker implantations was comparably low in both groups. However, atrial flutter was observed in two patients in the group of patients with PVI only. This might be related to the absence of a mitral isthmus line in those patients, begetting peri-mitral flutter, which is, however, a rare entity in patients with paroxysmal AF. All patients with atrial flutter were successfully treated with catheter-based ablations during 1-year follow-up.

## 5. Conclusions

In this selected patient collective that only included patients with paroxysmal AF undergoing CABG or AVR, a pulmonary vein isolation might be sufficient to achieve satisfactory rhythm results during one-year follow-up. Furthermore, no differences between complication and survival rates were observed between groups.

## 6. Limitations

This study is a retrospective one with the potential risk of bias by unknown confounders. Furthermore, our series is a single-center study and is limited by the heterogeneity of our patient population, including different ablation energy sources. Additionally, the follow-up was limited to 12 months.

## Figures and Tables

**Table 1:** Patient baseline characteristics.

	PVI *n* = 182	Ext. LA *n* = 86	*p* =
Age (years)	67.4 ± 6.5	66.0 ± 8.6	0.51
Gender (male *n*, (%))	115 (63.1)	51 (59.3)	0.23
LA diameter (mm)	52.5 ± 6.1	53.2 ± 6.7	0.12
AF duration (years)	2.7 ± 3.5	3.0 ± 2.3	0.25
LVEF (%)	54.8 ± 7.9	54.3 ± 6.5	0.10
Prior stroke *n* (%)	9 (4.9)	4 (5.8)	0.43
Renal insufficiency *n* (%)	12 (6.5)	5 (5.8)	0.35
Coronary artery disease	120 (65.9)	55 (63.9)	0.16

## Data Availability

Data can be made available upon reasonable request from the corresponding author.

## References

[1] Flaker G.C., Belew K., Beckman K., Vidaillet H., Kron J., Safford R., Mickel M., Barrell P., Affirm Investigators (2005). Asymptomatic atrial fibrillation: Demographic features and prognostic information from the Atrial Fibrillation Follow-up Investigation of Rhythm Management (AFFIRM) study. Am. Heart J..

[2] Fuster V., Ryden L.E., Cannom D.S., Crijns H.J., Curtis A.B., Ellenbogen K.A., Halperin J.L., Kay G.N., Le Hueze J.-Y., Lowe J.E. (2011). 2011 ACCF/AHA/HRS focused updates incorporated into the ACC/AHA/ESC 2006 Guidelines for the management of patients with atrial fibrillation: A report of the American College of Cardiology Foundation/American Heart Association Task Force on Practice Guidelines developed in partnership with the European Society of Cardiology and in collaboration with the European Heart Rhythm Association and the Heart Rhythm Society. J. Am. Coll. Cardiol..

[3] Phan K., Xie A., La Meir M., Black D., Yan T.D. (2014). Surgical ablation for treatment of atrial fibrillation in cardiac surgery: A cumulative meta-analysis of randomised controlled trials. Heart.

[4] Barnett S.D., Ad N. (2006). Surgical ablation as treatment for the elimination of atrial fibrillation: A meta-analysis. J. Thorac. Cardiovasc. Surg..

[5] Badhwar V., Rankin J.S., Ad N., Grau-Sepulveda M., Damiano R.J., Gillinov A.M., McCarthy P.M., Thourani V.H., Suri R.M., Jacobs J.P. (2017). Surgical Ablation of Atrial Fibrillation in the United States: Trends and Propensity Matched Outcomes. Ann. Thorac. Surg..

[6] Doukas G., Samani N.J., Alexiou C., Oc M., Chin D.T., Stafford P.G., Ng L.L., Spyt T.J. (2005). Left atrial radiofrequency ablation during mitral valve surgery for continuous atrial fibrillation: A randomized controlled trial. JAMA.

[7] Gillinov A.M., Gelijns A.C., Parides M.K., DeRose J.J., Moskowitz A.J., Voisine P., Ailawadi G., Bouchard D., Smith P.K., Mack M.J. (2015). Surgical ablation of atrial fibrillation during mitral-valve surgery. N. Engl. J. Med..

[8] Lee R., McCarthy P.M., Wang E.C., Vaduganathan M., Kruse J., Malaisrie S.C., McGee E.C. (2012). Midterm survival in patients treated for atrial fibrillation: A propensity-matched comparison to patients without a history of atrial fibrillation. J. Thorac. Cardiovasc. Surg..

[9] Musharbash F.N., Schill M.R., Sinn L.A., Schuessler R.B., Maniar H.S., Moon M.R., Melby S.J., Damiano R.J. (2018). Performance of the Cox-maze IV procedure is associated with improved long-term survival in patients with atrial fibrillation undergoing cardiac surgery. J. Thorac. Cardiovasc. Surg..

[10] Calkins H., Hindricks G., Cappato R., Kim Y.H., Saad E.B., Aguinaga L., Akar J.G., Badhwar V., Brugada J., Camm J. (2018). 2017 HRS/EHRA/ECAS/APHRS/SOLAECE expert consensus statement on catheter and surgical ablation of atrial fibrillation. Europace: European pacing, arrhythmias, and cardiac electrophysiology. J. Work. Groups Card. Pacing Arrhythm. Card. Cell. Electrophysiol. Eur. Soc. Cardiol..

[11] Badhwar V., Rankin J.S., Damiano R.J., Gillinov A.M., Bakaeen F.G., Edgerton J.R., Philpott J.M., McCharty P.M., Bolling S.F., Roberts H.G. (2017). The Society of Thoracic Surgeons 2017 Clinical Practice Guidelines for the Surgical Treatment of Atrial Fibrillation. Ann. Thorac. Surg..

[12] Cox J.L. (1991). The surgical treatment of atrial fibrillation. IV. Surgical technique. J. Thorac. Cardiovasc. Surg..

[13] Haissaguerre M., Jais P., Shah D.C., Takahashi A., Hocini M., Quiniou G., Garrigue S., Le Mouroux A., Le Métayer P., Clémenty J. (1998). Spontaneous initiation of atrial fibrillation by ectopic beats originating in the pulmonary veins. N. Engl. J. Med..

[14] Cosedis Nielsen J., Johannessen A., Raatikainen P., Hindricks G., Walfridsson H., Kongstad O., Pehrson S., Englund A., Hartikainen J., Mortensen L.S. (2012). Radiofrequency ablation as initial therapy in paroxysmal atrial fibrillation. N. Engl. J. Med..

[15] Vogler J., Willems S., Sultan A., Schreiber D., Luker J., Servatius H., Schäffer B., Moser J., Hoffmann B.A., Steven D. (2015). Pulmonary Vein Isolation Versus Defragmentation: The CHASE-AF Clinical Trial. J. Am. Coll. Cardiol..

[16] Genev I.K., Tompkins L.A., Khare M.M., Farokhi F. (2017). Comparison of the Efficancy and Complication Rates of the Hybrid Maze, Complete Cox Maze and Catheter Ablation in the Treatment of Atrial Fibrillation. J. Atr. Fibrillation.

[17] Gillinov A.M., Bhavani S., Blackstone E.H., Rajeswaran J., Svensson L.G., Navia J.L., Pettersson B.G., Sabik J.F., Smedira N.G., Mihaljevic T. (2006). Surgery for permanent atrial fibrillation: Impact of patient factors and lesion set. Ann. Thorac. Surg..

[18] Abreu Filho C.A., Lisboa L.A., Dallan L.A., Spina G.S., Grinberg M., Scanavacca M., Sosa E.A., Ramires J.A.F., Oliveira S.A. (2005). Effectiveness of the maze procedure using cooled-tip radiofrequency ablation in patients with permanent atrial fibrillation and rheumatic mitral valve disease. Circulation.

[19] Gelsomino S., La Meir M., Van Breugel H.N., Renzulli A., Rostagno C., Lorusso R., Parise O., Lozekoot P.W.J., Klop I.D.G., Kumar N. (2015). Surgical ablation in patients undergoing mitral valve surgery: Impact of lesion set and surgical techniques on long-term success. Europace: European pacing, arrhythmias, and cardiac electrophysiology. J. Work. Groups Card. Pacing Arrhythm. Card. Cell. Electrophysiol. Eur. Soc. Cardiol..

[20] McCarthy P.M., Manjunath A., Kruse J., Andrei A.C., Li Z., McGee E.C., Malaisrie S.C., Lee R. (2013). Should paroxysmal atrial fibrillation be treated during cardiac surgery?. J. Thorac. Cardiovasc. Surg..

[21] Soni L.K., Cedola S.R., Cogan J., Jiang J., Yang J., Takayama H., Argenziano M. (2013). Right atrial lesions do not improve the efficacy of a complete left atrial lesion set in the surgical treatment of atrial fibrillation, but they do increase procedural morbidity. J. Thorac. Cardiovasc. Surg..

[22] Nuzzi V., Cannatà A., Manca P., Castrichini M., Barbati G., Aleksova A., Fabris E., Zecchin M., Merlo M., Boriani G. (2021). Atrial fibrillation in dilated cardiomyopathy: Outcome prediction from an observational registry. Int. J. Cardiol..

